# Autologous liver transplantation for unresectable hepatobiliary malignancies in enhanced recovery after surgery model

**DOI:** 10.1515/med-2024-0926

**Published:** 2024-04-01

**Authors:** Weifeng Liu, Guogang Li, Yitian Jin, Yihui Feng, Zhenzhen Gao, Xingyu Liu, Bo Zhou, Xiang Zheng, Xiangru Pei, Yulian Ying, Qian Yu, Sheng Yan, Chenlu Hu

**Affiliations:** Department of Hepatobiliary and Pancreatic Surgery, The Second Affiliated Hospital, Zhejiang University School of Medicine, Hangzhou, China; Key Laboratory of Precision Diagnosis and Treatment for Hepatobiliary and Pancreatic Tumor of Zhejiang Province, Hangzhou, China; Nursing Department, The Second Affiliated Hospital of Zhejiang University School of Medicine, Hangzhou, China

**Keywords:** enhanced recovery after surgery, autologous liver transplantation, *ex vivo* liver resection, liver cancer

## Abstract

*Ex vivo* liver resection combined with autologous liver transplantation offers the opportunity to treat otherwise unresectable hepatobiliary malignancies and has been applied in clinic. The implementation of enhanced recovery after surgery (ERAS) program improves the outcome of surgical procedures. This is a retrospective single-center study including 11 cases of patients with liver cancer that underwent autologous liver transplantation and received ERAS: cholangiocarcinoma of the hilar region (*n* = 5), intrahepatic cholangiocarcinoma (*n* = 3), gallbladder cancer (*n* = 1), liver metastasis from colorectal cancer (*n* = 1), and liver metastasis from gastrointestinal mesenchymal tumor (*n* = 1). There were no deaths within 30 days and major complications occurred in two patients, and four patients were readmitted upon the first month after the surgery. Median hospital stay was 20 days (range 13–44) and median open diet was Day 4 (range 2–9) after surgery and median early post-operative activity was Day 5 (range 2–9) after surgery. In conclusion, autologous liver transplantation is feasible in the treatment of otherwise unresectable hepatobiliary malignancies, and our study showed favorable results with autologous liver transplantation in ERAS modality. ERAS modality provides a good option for some patients whose tumors cannot be resected *in situ* and offers a chance for rapid recovery.

## 1 Introduction

Surgical R0 resection is the preferred and most desirable treatment for liver malignancies because no gross or microscopic tumor remains in the primary tumor after resection [1,2]. Advanced hepatobiliary surgery techniques have been increasingly used for resection and revascularization of cases with large vessel involvement [3–5]. However, some tumors are still not easily removed. Therefore, alternative treatment methods such as allogeneic liver transplantation are needed for liver cancer, although the need for long-term immunosuppression and the shortage of organs have hindered the widespread use of allogeneic liver transplantation [6]. In 1988, Pichlmayr et al. first reported *ex vivo* liver resection and autologous liver transplantation for liver tumors [7]. *Ex vivo* liver resection can further expand the limits of resectability, which allows for the treatment of some otherwise unresectable hepatobiliary malignancies [8,9]. Autologous liver transplantation avoids the use of immunosuppressive drugs and could significantly improve surgical outcomes. In addition, it is not affected by current shortage of liver donors. Therefore, for conventionally unresectable hepatobiliary malignancies, *ex vivo* liver resection combined with autologous liver transplantation may offer a great treatment option [8,9].

The concept of enhanced recovery after surgery (ERAS) was developed in the 1990s initially for patients who had undergone colorectal surgery [10,11]. Since then, ERAS has been implemented in various surgical specialties [12–14]. Recent studies have shown that ERAS protocols used in liver surgery and liver transplantation can reduce the length of hospital and ICU stay [15–18]. In this study, we aimed to investigate whether autologous liver transplantation in the ERAS modality could improve postoperative outcomes. We reported the experience of autologous liver transplantation in the ERAS model in our center to assess perioperative morbidity and long-term outcomes.

## 2 Patients and methods

### 2.1 Study population and data collection

Exclusion criteria were (1) advanced age (≥70 years old), (2) being treated in ICU, (3) multiple organ failure, and (4) poor physical condition and other conditions unsuitable for carrying out ERAS. The inclusion criteria were dynamic, and we encouraged patients who had liver cancer and underwent autologous liver transplantation to participate as much as possible. Even patients with a history of multiple organ failure were recommended to enroll in ERAS programs when their condition improves or they are transferred from the ICU to the general ward. Eleven cases were retrospectively analyzed who underwent autologous liver transplantation between December 2021 and February 2023 at the Second Affiliated Hospital of Zhejiang University School of Medicine. Patients included cases with intra- and extra-hepatic cholangiocarcinoma, gallbladder cancer, and liver metastases. Data were collected from the medical charts of the patients and informed consent form was signed for each case.


**Ethics committee approval and patient consent:** The study was in accordance with the ethical guidelines of the 1975 Declaration of Helsinki. Ethical approval was obtained from the Ethics Committee of the Second Affiliated Hospital, School of Medicine, Zhejiang University.

### 2.2 Description of ERAS measures

**Figure 1:**
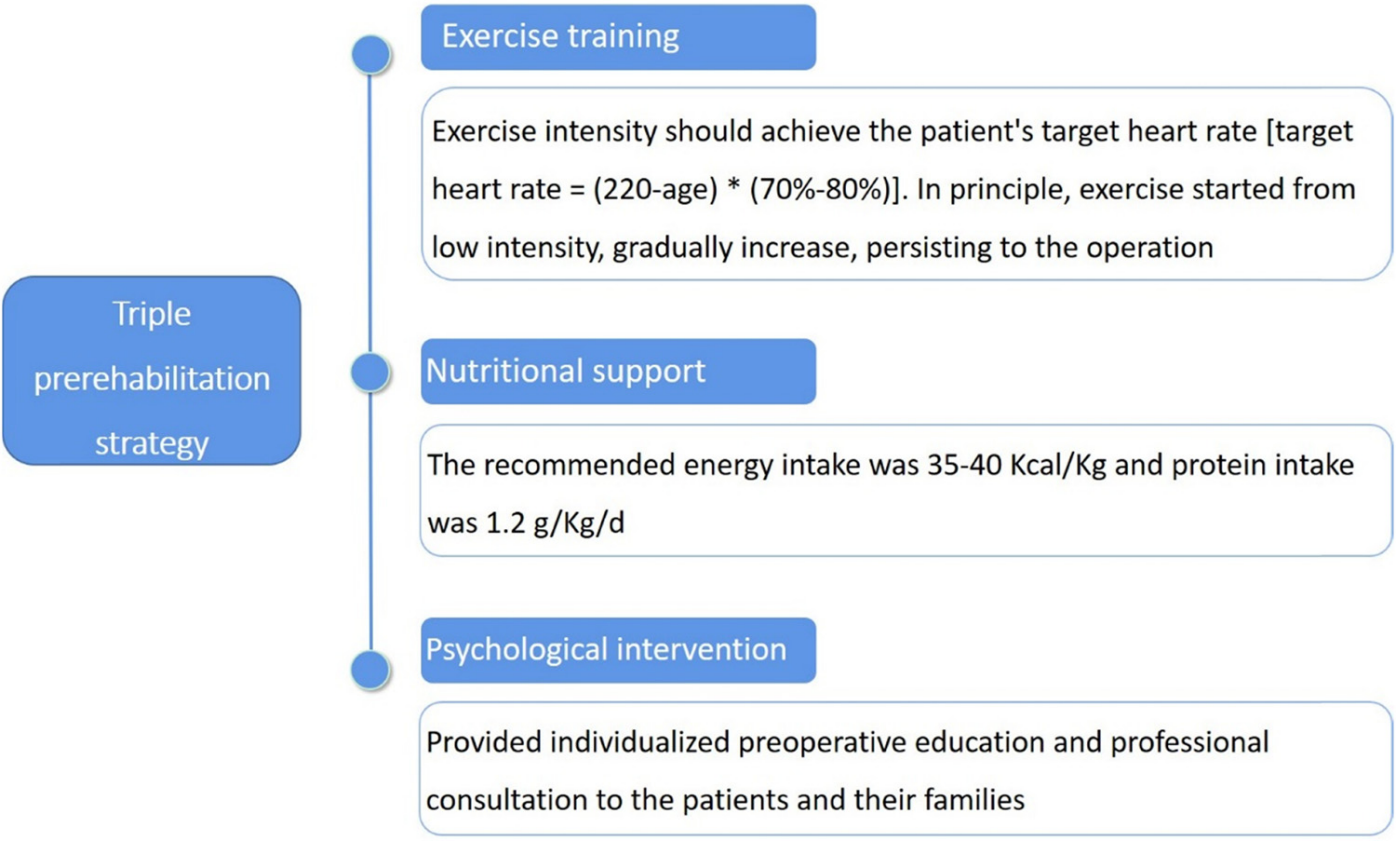
Summary of triple pre-rehabilitation strategy.

The triple pre-rehabilitation strategy, i.e., medium and high-intensity aerobic and strength exercise, nutritional support based on protein supplementation, and psychological support at present, was applied to eliminate anxiety during the waiting period for surgery (Figure 1). Preoperative exercise therapy plays a crucial role in enhancing patients’ cardiovascular and respiratory function, thereby creating optimal conditions for surgery and postoperative recovery. Supplementing protein before surgery fosters effective wound healing, muscle tissue repair, and boosts immunity. Additionally, psychological intervention aids in alleviating anxiety and negative emotions, consequently reducing postoperative fatigue.

The specific measures were as follows: (1) Exercise training: exercise intensity should achieve target heart rate: (220 − age) × (70–80%). Exercise training included rapid walking in the corridor of the department for 30 min at a target distance, twice a day; resistance exercise in the ward, lifting dumbbells for 15–20 min, once a day. At the same time, respiratory exercise was performed using a respiratory function exercise device, whose target of vital capacity was 2,650 mL, 15 min each time, three times a day; and effective cough exercise was 5–10 min once a day. After 8 days of preoperative exercise, the patient’s physical function was in good condition. (2) Nutritional support: The nutritional status of patients was evaluated with clinical dietitians. According to the expert consensus on enteral and parenteral nutrition support and dietary intervention for patients with chronic liver disease, the recommended energy intake was 35–40 kcal/kg and protein intake was 1.2 g/kg/day. (3) Psychological intervention: for surgical patients, early preoperative education and relaxation therapy were carried out to relieve tension. After the patients were admitted to the hospital, the responsible nurses provided individualized preoperative education and professional consultation to the patients and their families through videos, educational materials, and oral explanations. The patients were in a stable state of mind, sleeping normally, and actively cooperated with the work before operation.

**Figure 2:**
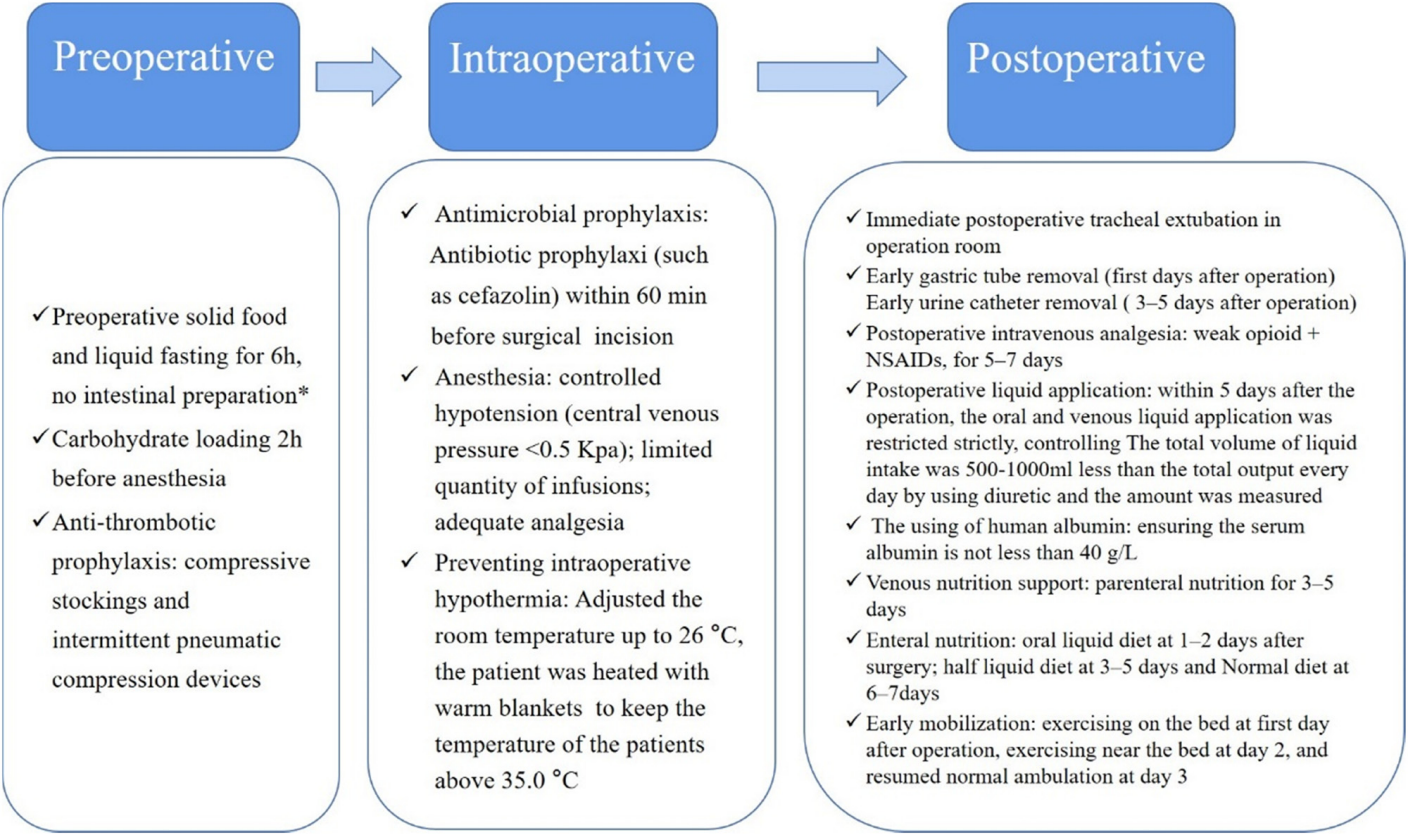
Summary of ERAS recommendations. * Indicates that if gastrointestinal surgery is performed, intestinal preparation is necessary.

Our department has implemented personalized care based on mind mapping for patients after autologous liver transplantation (Figure 2). (1) The patients were comprehensively evaluated by the multidisciplinary team before operation. Doctors and nurses cooperated to reach a consensus and formulate a personalized nursing plan. On the basis of routine nursing, we focused on the nursing of complications and effectively carried out personalized postoperative nursing under the mind map model. The mind map was drawn according to the patient’s nervous, digestive, circulatory, metabolic, and blood system, and focused attention and feedback on liver function, intake and output volume, drug concentration, etc. (2) According to the content of mind map, the department used collective teaching and online self-study to explain the related links of prevention and control of complications after autologous liver transplantation, and carried out specialized nursing operation assessment and team emergency drill at the same time. Through the learning of mind map, patients were comprehensively evaluated and analyzed. (3) Mind map is to sort out and compress the content, retain key information, help nurses quickly remember and accumulate knowledge points, effectively improve the quality of nursing, and reduce postoperative complications.

### 2.3 Statistical analysis

Quantitative variables were described using the median and range or the mean and standard deviation. Qualitative variables were described using percentages. Patient’s survival was estimated using the method of Kaplan–Meier.

## 3 Results

Demographic characteristics, patient history, and tumor type are shown in Table 1. The median age of the patients was 66 years (range 46–69) and eight patients (72.7%) were male. All patients had a Child-Pugh classification of A. Of the 11 patients, nine patients (81.8%) had primary cancer and two patients (18.2%) had metastatic cancer. Among the primary tumors, five cases were hilar cholangiocarcinoma, three cases were intrahepatic cholangiocarcinoma, and one case was a gallbladder carcinoma invading the hilar region. Two of the 11 cases were metastatic liver cancer, including one case of colorectal cancer liver metastasis and one case of gastrointestinal mesenchymal tumor liver metastasis. The number of tumors and the maximum tumor diameter for each patient are given in Table 2.

**Table 1:** Demographics and type of tumor

Age (years)	66 (46–69)
Male/female	8 (72.7)/3 (27.3)
Viral hepatitis history	1 (9.1)
Child-Pugh classification A/B/C	11 (100)/0/0
**Primary cancer**	
Intrahepatic cholangiocarcinoma	3
Hilar cholangiocarcinoma	5
Gallbladder carcinoma	1
**Metastatic liver cancer**	
Colorectal cancer liver metastasis	1
Gastrointestinal mesenchymalTumor liver metastasis	1

Number (percentage).

**Table 2:** Characteristics of the tumors

Type of tumor	Number of tumors	Maximum tumor diameter (cm)
Intrahepatic cholangiocarcinoma	1	4.0
Intrahepatic cholangiocarcinoma	1	6.5
Intrahepatic cholangiocarcinoma	1	6.0
Hilar cholangiocarcinoma	1	5.5
Hilar cholangiocarcinoma	2	3.0/2.7
Hilar cholangiocarcinoma	1	2.5
Hilar cholangiocarcinoma	1	3.0
Hilar cholangiocarcinoma	1	2.5
Gallbladder carcinoma	1	3.5
Colorectal cancer liver metastasis	1	6.0
Gastrointestinal mesenchymal tumor liver metastasis	1	20

According to statistical data, intrahepatic cholangiocarcinoma (iCCA) accounts for approximately 10–20% of cases, hilar cholangiocarcinoma (pCCA) for 50–60%, and extrahepatic cholangiocarcinoma for 20–30% among all cholangiocarcinoma cases. Autologous liver transplantation primarily targets patients with tumors in the hilar bile duct and intrahepatic bile duct, which invade the inferior vena cava and cannot be feasibly resected using conventional surgical methods. Thus, the selection of five cases of primary pCCA, three cases of iCCA, and one case of gallbladder carcinoma is consistent with tumor characteristics. Additionally, the inclusion of metastatic tumor cases was introduced to enhance the credibility of this study.

**Figure 3:**
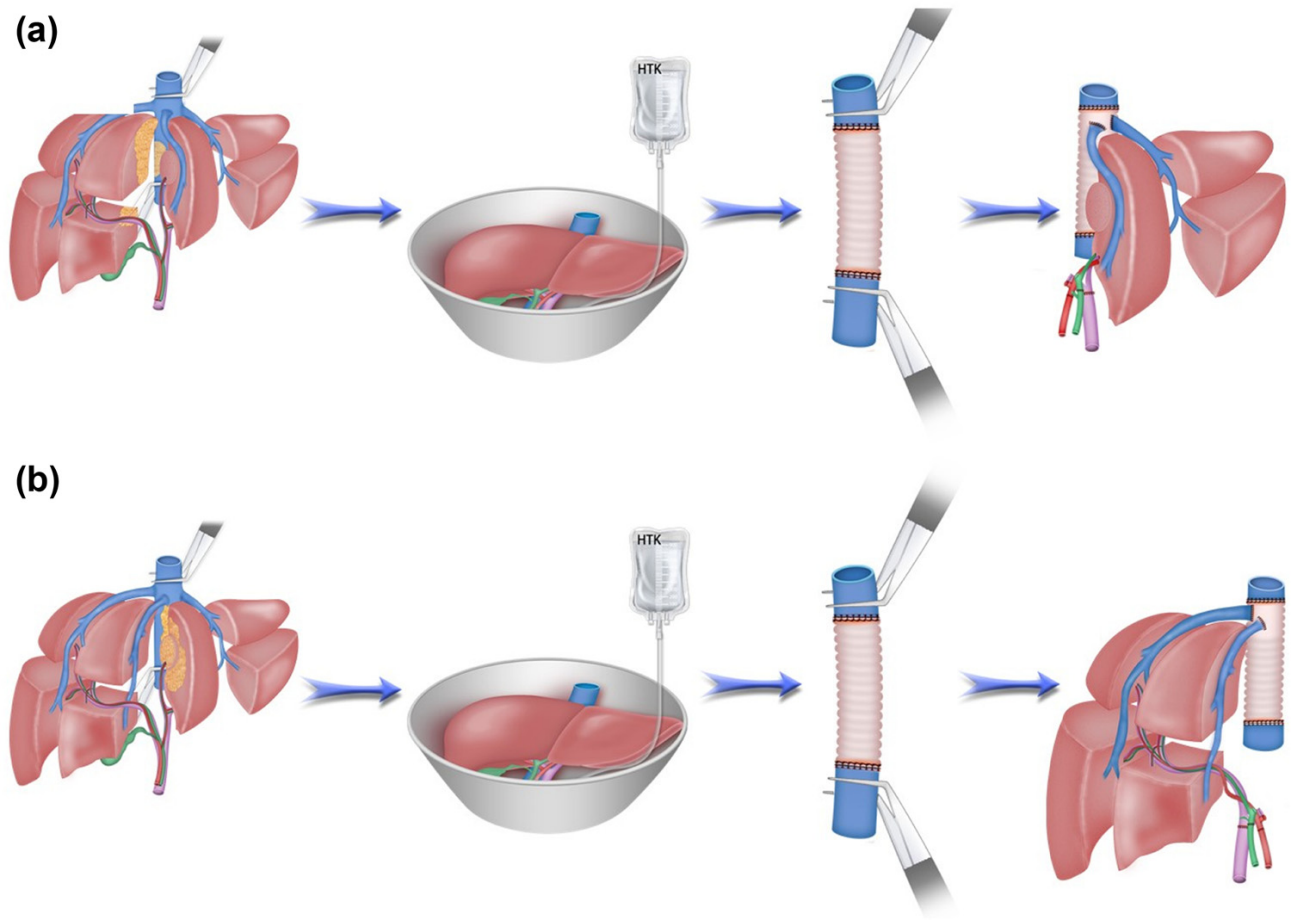
A schematic diagram of *in vivo* hepatectomy followed by *ex vivo* liver resection and autotransplantation. (a) The tumor was resected at the back table and an autograft of the left liver was created, artificial blood vessel replacement of the inferior vena cava was performed. (b) The tumor was resected at the back table and an autograft of the right liver was prepared, artificial blood vessel replacement of the inferior vena cava was performed.

The schematic diagram of *in vivo* hepatectomy followed by *ex vivo* liver resection and autologous liver transplantation are shown in Figure 3. Intraoperative and postoperative results are given in Table 3. In addition to autologous liver transplantation, two of the 11 patients underwent pancreaticoduodenectomy. Excluding these two patients, the median operative time for the remaining nine cases was 735 min. The mean surgical bleeding was 872 mL. Total two patients (18.2%) with hilar cholangiocarcinoma developed major postoperative complications, presented biliary leakage. The median hospital stay was 20 days (range 13–44). There were no deaths within 30 days among the 11 patients. Four of the 11 patients were readmitted within 30 days for right chest wall debridement and suturing, abnormal liver function and pleural effusion, ultrasound-guided percutaneous liver tumor microwave ablation, biliary tract infection, respectively.

**Table 3:** Intraoperative and postoperative results and ERAS-related measures

Operative time (min)	740 (550–890)
Bleeding volume (mL)	872
Postoperative complications	
Biliary leakage	2 (18.2)
Hospital stay (days)	20 (13–44)
Deaths within 30 days	0
Readmission within 30 days	4
Start early activity	5th (2nd–10th)
Open diet	4th (2nd–9th)

Number (percentage).

Ten of the 11 patients (90.9%) are currently alive with a median follow-up of 221 days (range 58–486). With regard to ERAS, the median time to start early activity was the fifth day after surgery (range 2nd–10th). The median time to open diet was the fourth day after surgery (range 2nd–9th). Drainage tube was placed in each case. The earliest removal of the drain was on the sixth postoperative day.

## 4 Discussion

The prognosis of initially unresectable hepatocellular carcinoma is very poor and how to improve treatment outcomes for the patients remains a great challenge in the clinic [19,20]. Traditional rehabilitation measures mostly start after surgery, but patients face problems such as decreased physical function, wound pain, anxiety or depression, and continue to receive radiotherapy or chemotherapy, which often lead to delayed rehabilitation and affecting recovery [13]. Pre-rehabilitation is an emerging preoperative management strategy based on ERAS, which emphasizes the optimization of patients’ functional ability and improvement of physiological function reserve, so as to better withstand surgical stress, accelerate postoperative functional recovery, and improve prognosis [14,18]. At present, we advocate the triple pre-rehabilitation strategy, i.e., medium and high-intensity aerobic and strength exercise, nutritional support based on protein supplementation, and psychological support to eliminate anxiety during the waiting period for surgery. The application of triple pre-rehabilitation strategy in liver surgery is safe and effective, which can significantly improve the perioperative activity ability, psychological status, and nutritional status of patients, reducing postoperative complications and accelerating postoperative rehabilitation. A systematic review revealed that liver transplant patients who receive ERAS strategies have superior outcomes compared to those receiving traditional rehabilitation strategies, including reduced overall postoperative complications, shorter stays in the intensive care unit, and shorter hospital stays [21]. In recent years, there have been several guidelines regulating ERAS protocols for liver transplantation, but more robust evidence is needed [22,23].

The surgical technique of autologous liver transplantation has become mature, and the effect is stable [3]. However, there are still some complications such as postoperative infection, postoperative bleeding, and biliary complications, which may affect the long-term survival of patients [24,25]. Following allograft liver transplantation, 78% of patients experienced at least one complication, with 54% experiencing multiple complications, and 63% requiring intervention for serious complications (≥grade 3) [26]. Regarding biliary complications post-liver transplantation, approximately 1–25% of patients may experience biliary leakage following allogeneic liver transplantation [27]. Data from our center demonstrated that the incidence of postoperative biliary complications in patients undergoing autologous liver transplantation in ERAS group did not significantly differ from previous report, and the overall complication rate was lower compared to the allograft group.

Therefore, it is of great importance for improving the prognosis and quality of life of patients to improve the postoperative nursing quality and rehabilitation effect. The implementation of personalized nursing based on mind map for patients after autologous liver transplantation in our department can enhance the clinical thinking ability of medical and nursing team, improve the ability of predicting complications, reduce the risk of postoperative complications, and significantly improve the quality of medical care.

*Ex vivo* liver resection and autologous liver transplantation has made it possible to restore access to treatment for some otherwise unresectable hepatobiliary malignancies, expanding the range of people who can be treated [8,9]. This procedure requires complex surgical skills and experience in liver transplantation and hypothermic cold perfusion [28]. Briefly, the surgeons need master expertise in the following steps: first, a total hepatectomy is performed and the liver is placed in a sterile ice bath. Second, malignant lesions are completely removed *in vitro* and autografts with blood vessels are prepared. Third, the graft is autografted into the right hepatic fossa, and the blood vessels and biliary tract are reconstructed [28].

In this study we reported the experiences of 11 cases of autologous liver transplantation performed at our hospital during 2021–2023. All patients enrolled had a Child-Pugh classification of A. Good liver function ensures that patients can tolerate surgery because if the residual liver does not have sufficient function to support patient survival after surgery, the surgery is not indicated in the first place. To date, ten cases are still alive, with an overall survival rate of 90.9%. Considering the relatively small number of cases, a larger number of case series would be necessary in the future.

In order to obtain better post-operative results and faster recovery after autologous liver transplantation, we implemented ERAS concept in the pre- and post-operative period of autologous liver transplantation, and achieved good results. However, this study has limitations. First, this study is retrospective, which may introduce biases and limitations in data collection and analysis. Prospective studies are needed to confirm our conclusion. Second, our sample size is not large. In conclusion, autologous liver transplantation in the ERAS modality may provide a good treatment option for some patients whose tumors cannot be resected *in situ* and may offer a chance for rapid recovery.
